# Comparison of Postoperative Outcomes Between Intra-aortic Balloon Pump and Levosimendan in Patients Undergoing Coronary Artery Bypass Graft: A Systematic Review and Meta-Analysis

**DOI:** 10.7759/cureus.43627

**Published:** 2023-08-17

**Authors:** Marwah Al-Tekreeti, Lokeshwar Raaju Addi Palle, Hamza Asif, Muhammad Fahad Amin, Hemalatha Anam, Yoshitha Akurathi, Saima Batool, Neelum Ali

**Affiliations:** 1 Internal Medicine, Avalon University School of Medicine, Willemstad, CUW; 2 Surgery, Kamala Children's Hospital, Chennai, IND; 3 General Surgery, Hackensack Meridian Health - Palisades Medical Center, North Bergen, USA; 4 Pulmonology, Khyber Teaching Hospital, Peshawar, PAK; 5 Medicine, Ghurki Trust Teaching Hospital, Lahore, PAK; 6 Medicine, Jinnah Hospital, Lahore, PAK; 7 Medicine, Apollo Institute of Medical Sciences and Research, Hyderabad, IND; 8 Internal Medicine, Narayana Medical College, Nellore, IND; 9 Internal Medicine, Hameed Latif Hospital, Lahore, PAK; 10 Internal Medicine, University of Health Sciences, Lahore, PAK

**Keywords:** postoperative outcomes, systematic review and meta-analysis, levosimendan, intra-aortic balloon pump, coronary artery bypass graft surgery

## Abstract

This study was conducted to compare the postoperative outcomes between intra-aortic balloon pump (IABP) and levosimendan in patients undergoing coronary artery bypass graft (CABG) surgery. This meta-analysis was conducted following the recommendations of Preferred Reporting Items for Systematic Reviews and Meta-Analyses (PRISMA). For this meta-analysis, a literature search was performed on PubMed, Cochrane Central Register of Controlled Trials, and EMBASE from inception to July 15, 2023. Keywords used to search for relevant articles included "intra-aortic balloon," "levosimendan," and "cardiac surgery" along with their key terms and Medical Subject Headings (MeSH) terms. Outcomes assessed in this study included postoperative outcomes like all-cause mortality, postoperative arrhythmias, need for inotropic support, length of intensive care unit stay (ICU) in days, and duration of mechanical ventilation in hours. Other outcomes included two-hour, six-hour, and 24-hour postoperative central venous pressure (CVP), mean atrial pressure (MAP), and heart rate (HR). A total of eight studies were included in the pooled analysis. The pooled results found that the length of ICU stay and the duration of mechanical ventilation were significantly higher in patients receiving IABP. Additionally, the findings of this meta-analysis showed a higher need for inotropic support in patients receiving IABP compared to patients receiving levosimendan but the difference was statistically insignificant. However, no significant differences were found between the two groups in terms of mortality and arrhythmias. In conclusion, patients treated with levosimendan exhibited significant advantages, as they experienced shorter ICU stays and reduced duration of mechanical ventilation compared to the IABP group and less requirement for inotropic support.

## Introduction and background

In recent times, patients undergoing cardiac surgery are characterized by advanced age, a higher number of preoperative co-existing health conditions, and compromised left ventricular function, and are often referred to as 'high-risk' patients [1]. To mitigate the risks associated with these patients, an internal counterpulsation device known as the intra-aortic balloon pump (IABP) has been employed as a circulatory support aid [2]. When the IABP is inflated during diastole, it can potentially enhance blood flow to the brain, heart, and systemic circulation. The use of perioperative IABP insertion in cardiac surgery is well-documented and supported by existing literature [3].

The IABP has established itself as an effective supplementary therapy for patients with a failing heart after experiencing myocardial infarction, unstable angina, or cardiac surgery [4]. It improves the balance between myocardial oxygen supply and demand, reduces afterload, and raises diastolic pressure, all of which contribute to improved cardiac function. The elevation in diastolic pressure also facilitates better blood flow to ischemic regions of the heart muscle [5]. A study involving multiple medical centers found that prophylactic use of the IABP led to improved outcomes in high-risk cardiac patients [6]. However, a significant drawback of the IABP, particularly in patients with systemic atherosclerosis, is the possibility of complications related to the installation of the balloon, including limb ischemia, vessel damage, and bleeding [7].

Levosimendan is classified as a calcium sensitizer and works by increasing myocardial contractility without a simultaneous rise in oxygen consumption. Moreover, it exhibits certain cardioprotective properties during myocardial ischemia development [8]. This is attributed to the fact that it does not elevate total intracellular calcium levels. Levosimendan has no adverse impact on the duration of diastole, thereby preserving ventricular relaxation and ensuring adequate ventricular filling and optimal coronary perfusion. However, its vasodilatation effect, which occurs through the opening of potassium channels, may predispose patients to hypotension [9]. A notable advantage of levosimendan is its long-term benefits, as it produces a pharmacologically active metabolite with a prolonged elimination half-life (75-80 hours), leading to sustained hemodynamic effects lasting for up to seven to nine days [10]. This meta-analysis aims to compare IABP and levosimendan in cardiac surgery patients, addressing conflicting results from individual studies. By pooling estimates, it increases statistical power and precision. The comprehensive synthesis of evidence helps identify clinical significance, guiding clinicians in making informed decisions for improved patient outcomes. Therefore, this meta-analysis has been conducted to compare the postoperative outcomes between IABP and levosimendan in patients undergoing coronary artery bypass graft (CABG) surgery.

## Review

Methodology

This meta-analysis has been conducted following the recommendations of Preferred Reporting Items for Systematic Reviews and Meta-Analyses (PRISMA).

Search Strategy

For this meta-analysis, a literature search was performed on PubMed, Cochrane Central Register of Controlled Trials, and EMBASE, from inception to July 15, 2023. Keywords used to search for relevant articles included "intra-aortic balloon," "levosimendan," and "cardiac surgery," along with their key terms and Medical Subject Headings (MeSH) terms. The search was restricted to articles published in the English language. All articles obtained through online database searching were imported into EndNote X9. After removing duplicates, articles were initially screened based on their titles/abstracts, followed by full-text screening using pre-defined inclusion and exclusion criteria. The search and selection of studies were independently performed by two authors. Any disagreement was resolved through consensus.

Eligibility Criteria

We included studies that met each of the following PICOS (population, intervention, comparison, and outcomes) criteria:

Population: Adult patients (≥18 years) undergoing coronary artery bypass graft (CABG).

Intervention: Levosimendan, irrespective of the dosage.

Comparison intervention: Intra-aortic balloon pump (IABP).

Outcome: Postoperative outcomes included (all-cause mortality, post-operative arrhythmias, need for inotropic support, length of intensive care unit stay (ICU) in days, and duration of mechanical ventilation in hours. Other outcomes included two-hour, six-hour, and 24-hour postoperative central venous pressure (CVP), mean atrial pressure (MAP), and heart rate (HR).

Study design: Randomized controlled trials (RCTs) or observational studies.

Data Collection Process and Assessment of Risk of Bias

Two reviewers independently conducted data extraction using a standardized electronic form. In case of disagreements, a third reviewer intervened to resolve the discrepancies through discussion and adjudication. Data extracted from the studies included the first author's name, publication year, study design, study setting, sample size, participants' characteristics, and outcomes. The Cochrane Risk of Bias tool was used to assess the risk of bias in RCTs. The risk of bias for each domain was categorized as high, low, or unclear. For observational studies, the Newcastle-Ottawa scale was used, and the overall risk of bias for each study was assessed as good, fair, or poor. The risk of bias assessment was independently performed by two authors, and any disagreement in this process was resolved through discussion and adjudication.

Data Analysis

We used RevMan version 5.4 (Cochrane Collaboration, London, United Kingdom) to perform the data analysis. For dichotomous outcomes, we calculated the risk ratio (RR) with a 95% confidence interval (CI) using the Mantel-Haenszel (M-H) method, and for continuous outcomes, the mean difference (MD) was calculated with a 95% CI, with a p-value of less than 0.05 denoting statistical significance. We used the random-effects meta-analysis method, as it can account for the variation among the study results. We assessed statistical heterogeneity using I-square statistics and the Cochran-Q test. A p-value less than 0.1 for the Cochran-Q test was considered significant for heterogeneity.

Results

**Figure 1:**
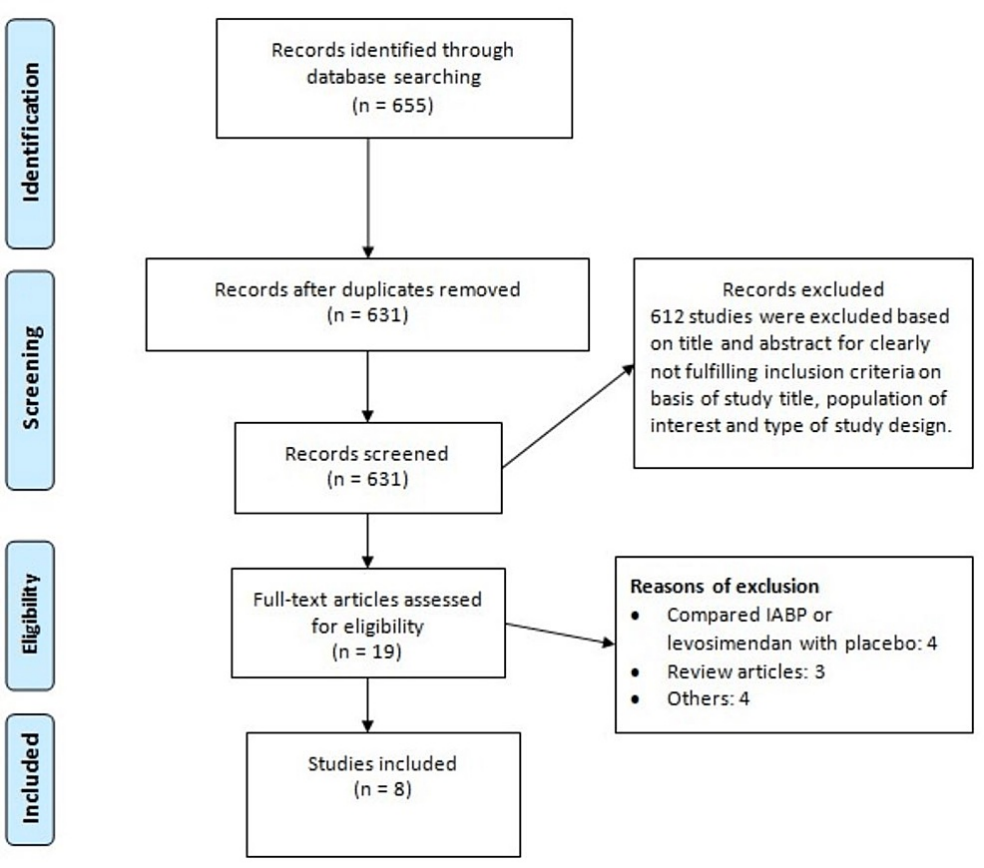
PRISMA flowchart PRISMA: Preferred Reporting Items for Systematic Reviews and Meta-Analyses

**Figure 2:**
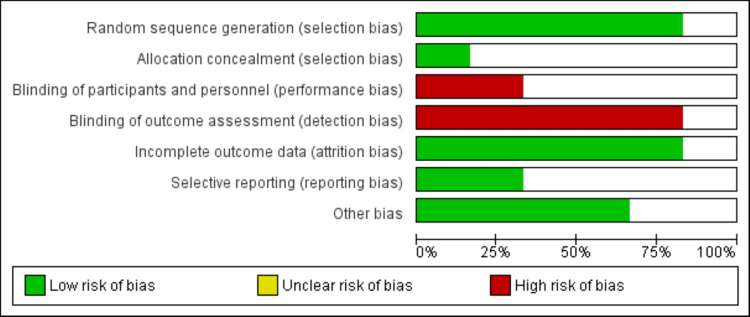
Risk of bias graph

The database search yielded 655 citations. After screening the titles and abstracts, 19 full-texts were screened and eight articles met the pre-defined eligibility criteria and were included in our meta-analysis. No further publications were obtained from the reference lists of the included studies. Figure 1 shows the PRISMA flowchart representing the study selection process. The characteristics of included studies are shown in Table 1. Among all included studies, six were RCTs and two were observational. The majority of participants in all included studies were males. Figure 2 shows the risk of bias assessment of included RCTs. Table 2 shows the quality assessment of observational studies.

**Table 1:** Characteristics of included studies RCT: Randomized control trial; EF: Ejection fraction; IABP: Intra-aortic balloon pump

Author	Year	Study Design	Groups	Sample Size	Mean Age	Males (%)	Mean EF
Allama et al [11]	2020	RCT	IABP	30	57.7	73	32.7
			Levosimendan	30	58.9	83	33.2
Azzab et al [12]	2021	Observational	IABP	30	58.4	87	33.1
			Levosimendan	30	57.7	80	43.2
Hady et al [13]	2021	RCT	IABP	30	53.3	67	30
			Levosimendan	30	61.9	66	33
Lomivorotov et al [14]	2011	RCT	IABP	20	57.7	85	29
			Levosimendan	20	57	80	29.8
Lomivorotov et al [15]	2012	RCT	IABP	30	56.8	97	30
			Levosimendan	30	57.3	80	31
Mate et al [16]	2020	RCT	IABP	30	61.2	70	20.4
			Levosimendan	30	60.2	77	20.5
Omar et al [17]	2019	RCT	IABP	144	58.8	83	33
			Levosimendan	135	57.7	73	32.2
Severi et al [18]	2011	Observational	IABP	11	60	90.9	26
			Levosimendan	11	66	90.9	30

**Table 2:** Quality assessment of observational studies

Study Id	Selection	Exposure	Outcome
Azzab et al [12]	3	2	3
Severi et al [18]	3	1	3

All-Cause Mortality

**Figure 3:**
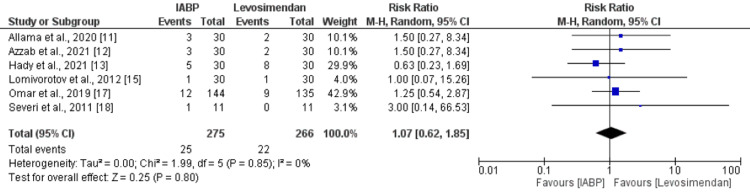
All-cause mortality IABP: Intra-aortic balloon pump Sources: [11-13,15,17-18]

Pooled analysis of six studies reported no significant difference in risk of all-cause mortality between the IABP and levosimendan groups (RR: 1.07, 95% CI: 0.62 to 1.85, p-value: 0.80) as shown in Figure 3. No significant heterogeneity was reported among the study results (I-square: 0%).

Postoperative Arrhythmias and Need for Inotropic Support

**Figure 4:**
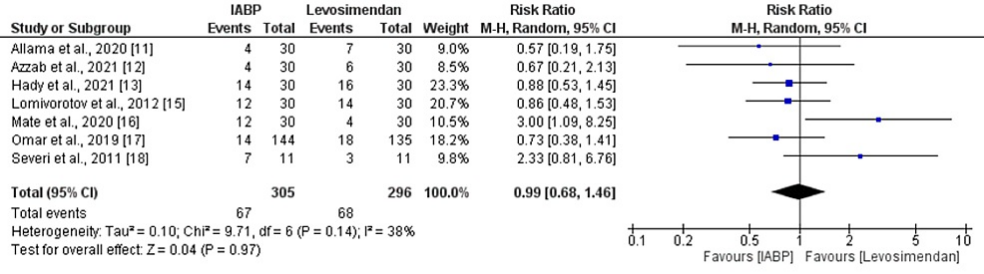
Postoperative arrhythmias IABP: Intra-aortic balloon pump Sources: [11-18]

**Figure 5:**
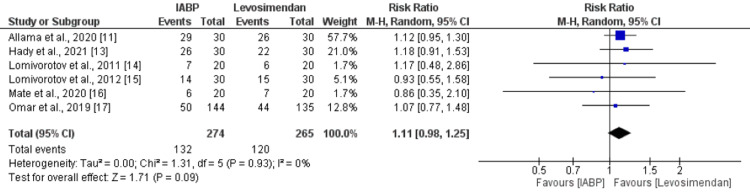
Need for inotropic support IABP: Intra-aortic balloon pump Sources: [11,13-17]

Eight studies were included in the pooled analysis of postoperative arrhythmia. The pooled analysis reported no significant difference between the IABP and levosimendan groups in terms of postoperative arrhythmias (RR: 0.99, 95% CI: 0.68 to 1.46, p-value: 0.97) as shown in Figure 4. No significant heterogeneity was reported among the study results (I-square: 38%). Similarly, in relation to inotropic support, six studies assessed this outcome. As shown in Figure 5, the need for inotropic support was higher in patients receiving IABP compared to levosimendan, but the difference was statistically insignificant (RR: 1.11, 95% CI: 0.98 to 1.25). No significant heterogeneity was reported among the study results (I-square: 0%).

Length of ICU Stay and Duration of Mechanical Ventilation

**Figure 6:**
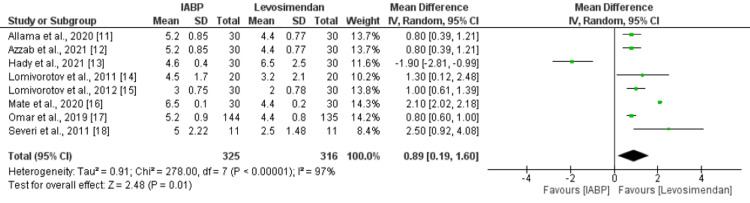
Length of ICU stay (days) IABP: intra-aortic balloon pump Sources: References [11-18]

**Figure 7:**
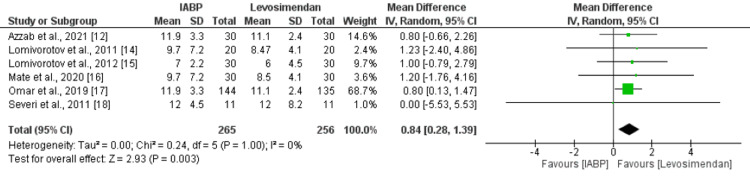
Duration of mechanical ventilation (hours) IABP: intra-aortic balloon pump Sources: [12,14-18]

Eight studies compared the length of ICU stay between the IABP and levosimendan groups. Figure 6 shows that the mean length of ICU stay was significantly higher in the IABP group as compared to the levosimendan group (MD: 0.89, 95% CI: 0.19 to 1.60, p-value: 0.01). Significant heterogeneity was reported among the study results (I-square: 97%). Pooled analysis of six studies showed the duration of mechanical ventilation was significantly higher in patients in the IABP group compared to the levosimendan group (MD: 0.84, 95% CI: 0.28 to 1.39, p-value: 0.003) as shown in Figure 7. No significant heterogeneity was reported among the study results (I-square: 0%).

Postoperative Hemodynamic Data

The postoperative hemodynamic results are presented in Table 3. Mean arterial pressure (MAP) was higher in the IABP group at two hours, six hours, and 24 hours postoperatively, but a significant difference was only observed at two hours. Central venous pressure (CVP) was significantly higher in patients in the levosimendan group compared to the IABP group at two hours and 24 hours after the surgery. Heart rate (HR) was also higher in the levosimendan group compared to the IABP group at all time points, but the differences were statistically insignificant.

**Table 3:** Postoperative hemodynamic data MD: Mean difference; CI: Confidence interval; HR: Heart rate; CVP: Central venous pressure; MAP: Mean arterial pressure * Significant at p-value <0.05

Time Duration	Outcomes	MD (95% CI)	I-square
2 Hours Postoperatively	HR	-1.18 (-5.86, 3.49)	68%
	MAP	5.14 (3.49, 6.79)*	0%
	CVP	-0.61 (-0.95, -0.27)*	0%
6 Hours Postoperatively	HR	-4.02 (-8.25, 0.20)	75%
	MAP	2.76 (-0.21, 5.73)	74%
	CVP	-0.80 (-2.21, 0.61)	91%
24 Hours Postoperatively	HR	-5.78 (-8.90, -2.66)*	70%
	MAP	1.11 (-3.16, 5.39)	89%
	CVP	-0.42 (-0.64, -0.21)*	0%

Discussion

This meta-analysis compared outcomes between IABP and levosimendan in patients undergoing cardiac surgery. The pooled results found that the length of ICU stay and the duration of mechanical ventilation were significantly higher in patients receiving IABP. Additionally, the findings of this meta-analysis have shown a higher need for inotropic support in patients receiving IABP compared to patients receiving levosimendan but the difference was statistically insignificant. However, no significant differences were found between the two groups in terms of mortality and arrhythmias.

The major challenge during cardiac surgery is to maintain optimal hemodynamics, which can be accomplished by pharmacological or mechanical means [19]. The utilization of inotropes represents a significant pharmacological intervention, and their careful selection contributes to improved clinical results. Nevertheless, conventional inotropes like beta-agonists and phosphodiesterase inhibitors are linked to tachycardia and arrhythmia, resulting in heightened myocardial oxygen demand [20]. Levosimendan, a new inotropic agent, offers not only enhanced contractility but also advantageous immunomodulatory, cardioprotective, anti-stunning, anti-ischemic, anti-inflammatory, and antioxidant effects. These combined benefits aim to enhance cardiac performance even under conditions of ischemia [21].

While numerous studies have demonstrated that employing IABP before CABG surgery in high-risk patients, particularly those with low LVEF, can enhance surgical outcomes by reducing in-hospital mortality, improving postoperative hemodynamic parameters, and shortening ICU stay, it is essential to acknowledge that IABP application is an invasive procedure with potential adverse effects such as limb ischemia and bleeding [18]. Levosimendan is one of the alternatives to IABP to maintain hemodynamics in patients undergoing cardiac surgery, and the current study has shown that no significant differences exist between the two groups in relation to hemodynamic measures, including MAP, HR, and CVP.

In a recent meta-analysis, Landoni et al. reported that administering levosimendan leads to a significant decrease in mortality among cardiac patients [22]. Wang et al. also demonstrated that IABP offers a reduction in short-term mortality in high-risk CABG patients [23], with a similar conclusion being reached by Suzuki and colleagues [24]. Our study findings corroborate these results, revealing that initiating levosimendan infusion after anesthesia induction serves as a favorable alternative to IABP in high-risk cardiac patients, as no significant differences were reported between the two groups in relation to major complications like mortality and arrhythmias.

The present study reported a shorter duration of ICU stay and mechanical ventilation in the levosimendan group compared to the IABP group. It also reduces the need for inotropes. This clearly shows the advantages of levosimendan over IABP in patients undergoing cardiac surgery. The current meta-analysis shows that levosimendan can be a viable alternative to IABP in high-risk cardiac surgery. Based on the authors' experience, initiating levosimendan infusion shortly after anesthesia induction without a loading dose yields comparable results to IABP. Patients with absolute contraindications for IABP, such as severe aortic dissection, aortic regurgitation, and severe peripheral arterial disease, are prime candidates for levosimendan-based management. Levosimendan may potentially replace the invasive use of IABP in certain categories of high-risk cardiac surgery patients, pending confirmation of these findings through additional studies. Notably, both levosimendan and IABP have been recognized in an international consensus conference as among the few drugs or techniques that can reduce mortality in cardiac surgery [25].

The current meta-analysis has certain limitations. First, the sample size of all included studies was low. Second, due to a lack of individual-level data that affect outcomes like all-cause mortality and arrhythmias, we were not able to perform subgroup analysis. Therefore, in the future, large-scale RCTs are required to confirm the findings of this study and to develop guidelines for using levosimendan in patients undergoing CABG.

## Conclusions

In conclusion, this comprehensive meta-analysis comparing the outcomes of IABP and levosimendan in patients undergoing cardiac surgery provides valuable insights into the effectiveness and safety of these interventions. The findings reveal that there is no significant difference in all-cause mortality between the two treatment groups, indicating that both IABP and levosimendan are comparable in terms of mortality risk. However, patients treated with levosimendan exhibited significant advantages, as they experienced shorter ICU stays and a reduced duration of mechanical ventilation compared to the IABP group and had less requirement of inotropic support. This indicates that levosimendan may offer better postoperative recovery and resource utilization in these patients. However, further large-scale RCTs are required to validate these findings.
